# Nano‐Regulator Inhibits Tumor Immune Escape via the “Two‐Way Regulation” Epigenetic Therapy Strategy

**DOI:** 10.1002/advs.202305275

**Published:** 2023-12-18

**Authors:** Shuang Liang, Meichen Liu, Weiwei Mu, Tong Gao, Shuying Gao, Shunli Fu, Shijun Yuan, Jinhu Liu, Yongjun Liu, Dandan Jiang, Na Zhang

**Affiliations:** ^1^ Department of Pharmaceutics Key Laboratory of Chemical Biology (Ministry of Education) School of Pharmaceutical Sciences Cheeloo College of Medicine Shandong University 44 Wenhua Xi Road Jinan Shandong 250012 China; ^2^ Department of Pharmacy Henan Provincial People's Hospital People's Hospital of Zhengzhou University Zhengzhou Henan 450003 China

**Keywords:** epigenetic regulation, nano‐regulator, tumor immune escape, two‐way regulation

## Abstract

Tumor immune escape caused by low levels of tumor immunogenicity and immune checkpoint‐dependent suppression limits the immunotherapeutic effect. Herein, a “two‐way regulation” epigenetic therapeutic strategy is proposed using a novel nano‐regulator that inhibits tumor immune escape by upregulating expression of tumor‐associated antigens (TAAs) to improve immunogenicity and downregulating programmed cell death 1 ligand 1 (PD‐L1) expression to block programmed death‐1 (PD‐1)/PD‐L1. To engineer the nano‐regulator, the DNA methyltransferase (DNMT) inhibitor zebularine (Zeb) and the bromodomain‐containing protein 4 (BRD4) inhibitor JQ1 are co‐loaded into the cationic liposomes with condensing the toll‐like receptor 9 (TLR9) agonist cytosine‐phosphate‐guanine (CpG) via electrostatic interactions to obtain G‐J/ZL. Then, asparagine–glycine–arginine (NGR) modified material carboxymethyl‐chitosan (CMCS) is coated on the surface of G‐J/ZL to construct CG‐J/ZL. CG‐J/ZL is shown to target tumor tissue and disassemble under the acidic tumor microenvironment (TME). Zeb upregulated TAAs expression to improve the immunogenicity; JQ1 inhibited PD‐L1 expression to block immune checkpoint; CpG promote dendritic cell (DC) maturation and reactivated the ability of tumour‐associated macrophages (TAM) to kill tumor cells. Taken together, these results demonstrate that the nano‐regulator CG‐J/ZL can upregulate TAAs expression to enhance T‐cell infiltration and downregulate PD‐L1 expression to improve the recognition of tumor cells by T‐cells, representing a promising strategy to improve antitumor immune response.

## Graphical abstract

The “two‐way regulation” epigenetic therapy strategy via designing an integrated nano‐regulator is proposed to inhibit tumor immune escape, which can upregulate tumor‐associated antigens (TAAs) expression to enhance T‐cell infiltration and downregulate PD‐L1 expression to improve the recognization of T‐cells to tumor cells, thus inhibiting tumor immune escape and activating antitumor immune response.

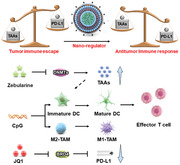

## 1 Introduction

Immunotherapy is one of the most effective methods for tumor treatment.^[^ ^1^, ^2^, ^3^, ^4^ ^]^ However, most patients do not benefit from immunotherapy, and the overall response rate of immunotherapy is only 10%–35%.^[^ ^5^, ^6^, ^7^, ^8^ ^]^ The immunoediting theory pointed out that immune escape is the biggest obstacle to immunotherapy,^[^ ^9^, ^10^ ^]^ including the low levels of tumor immunogenicity^[^ ^11^, ^12^, ^13^, ^14^ ^]^ and immune checkpoint‐dependent suppression,^[^ ^15^, ^16^ ^]^ which caused insufficient T‐cells intratumoral infiltration and insufficient recognization of tumor cells by T‐cells.^[^ ^17^ ^]^

Epigenetic regulation is an important reason for tumor immune escape.^[^ ^18^, ^19^ ^]^ DNA methylation is one of the main epigenetic regulations that occurs by the addition of a methyl (CH_3_) group to DNA under the catalysis of DNA methyltransferases (DNMT), which controls gene expression.^[^ ^20^, ^21^, ^22^ ^]^ It was reported that there was DNA hypermethylation in tumor‐associated antigens (TAAs) promoter regions, which caused the low level of tumor immunogenicity via reducing TAAs expression, thereby decreasing the ability of antigen‐presenting cells (APC) to present antigens to T‐cells^[^ ^23^ ^]^ Therefore, inhibition of DNA hypermethylation of tumor cells is expected to directly upregulate the expression of TAAs, which would increase tumor immunogenicity and enhance T‐cells intratumoral infiltration.

In addition to the epigenetic regulation of DNA hypermethylation to inhibit tumor antigen expression, tumor cells can also regulate the histone acetylation mechanism to control immune checkpoint expression. Programmeddeath‐1 (PD‐1) is a receptor that is primarily expressed on T‐cells and acts to reduce the immune response^[^ ^24^ ^]^ Tumor cells express a large number of programmed cell death 1 ligand 1 (PD‐L1) on the surface to help tumor cells escape immune surveillance by binding to the PD‐1 receptor on T‐cells^[^ ^25^ ^]^ Therefore, inhibiting the expression of PD‐L1 in tumor cells is an effective mean to block PD‐1/PD‐L1, thereby reactivating T‐cells to kill tumors. PD‐L1 is encoded by the CD274 gene that located on chromosome 9p24.1, and the changes in the chromatin structure and properties of 9p24.1 directly affect the expression of PD‐L1^[^ ^26^ ^]^ The bromodomain and extra terminal domain (BET) protein family is an epigenetic reader for histone acetylation, which can regulate gene transcription^[^ ^27^ ^]^ Bromine domain protein 4 (BRD4), a member of the BET protein family, is mainly involved in the expression of PD‐L1, which can bind to the acetylated histone H3K27Ac in the CD274 promoter and enhancer region to promote the expression of PD‐L1.^[^ ^28^, ^29^ ^]^ Therefore, the use of BDD4 inhibitors can effectively block the PD‐1/PD‐L1 immune checkpoint, thereby reactivating the killing of tumor cells by immune effector cells.

Based on the significant influence of epigenetic regulations on tumor immune escape, a “two‐way regulation” epigenetic therapy strategy was first proposed to inhibit tumor immune escape via an integrated nano‐regulator, which can upregulate TAAs expression to improve the immunogenicity and downregulate PD‐L1 expression to block the PD‐1/PD‐L1 immune checkpoint. To engineer the nano‐regulator, the DNMT inhibitor zebularine (Zeb)^[^ ^30^, ^31^, ^32^ ^]^ and the BRD4 inhibitor JQ1^[^ ^29^ ^]^ were co‐loaded into cationic liposomes with condensing Toll‐like receptor 9 (TLR9) agonist CpG^[^ ^33^, ^34^, ^35^, ^36^ ^]^ (G‐J/ZL) via electrostatic interaction, and then the targeting ligand asparagine–glycine–arginine (NGR)^[^ ^37^ ^]^ modified charge reversal material carboxymethyl chitosan (CMCS) was coated on the surface of G‐J/ZL to construct a core‐shell structure CG‐J/ZL. Under the mediation of NGR, CG‐J/ZL could target tumor tissue and trigger disassembly under the acidic TME. Zeb could effectively upregulate TAAs on tumor surface by inhibiting DNA methyltransferases, thus enhancing tumor immunogenicity and improving immune recognition. JQ1 could inhibit PD‐L1 expression to block immune checkpoint blockade; CpG, as a TLR9 agonist, could promote dendritic cells (DC) maturation that would cooperate with Zeb to promote activation of T‐cells. Besides, CpG could “wake up” tumor‐associated macrophages (TAM), and reactivate the ability of TAM to kill tumor cells^[^ ^38^ ^]^

**Scheme 1:**
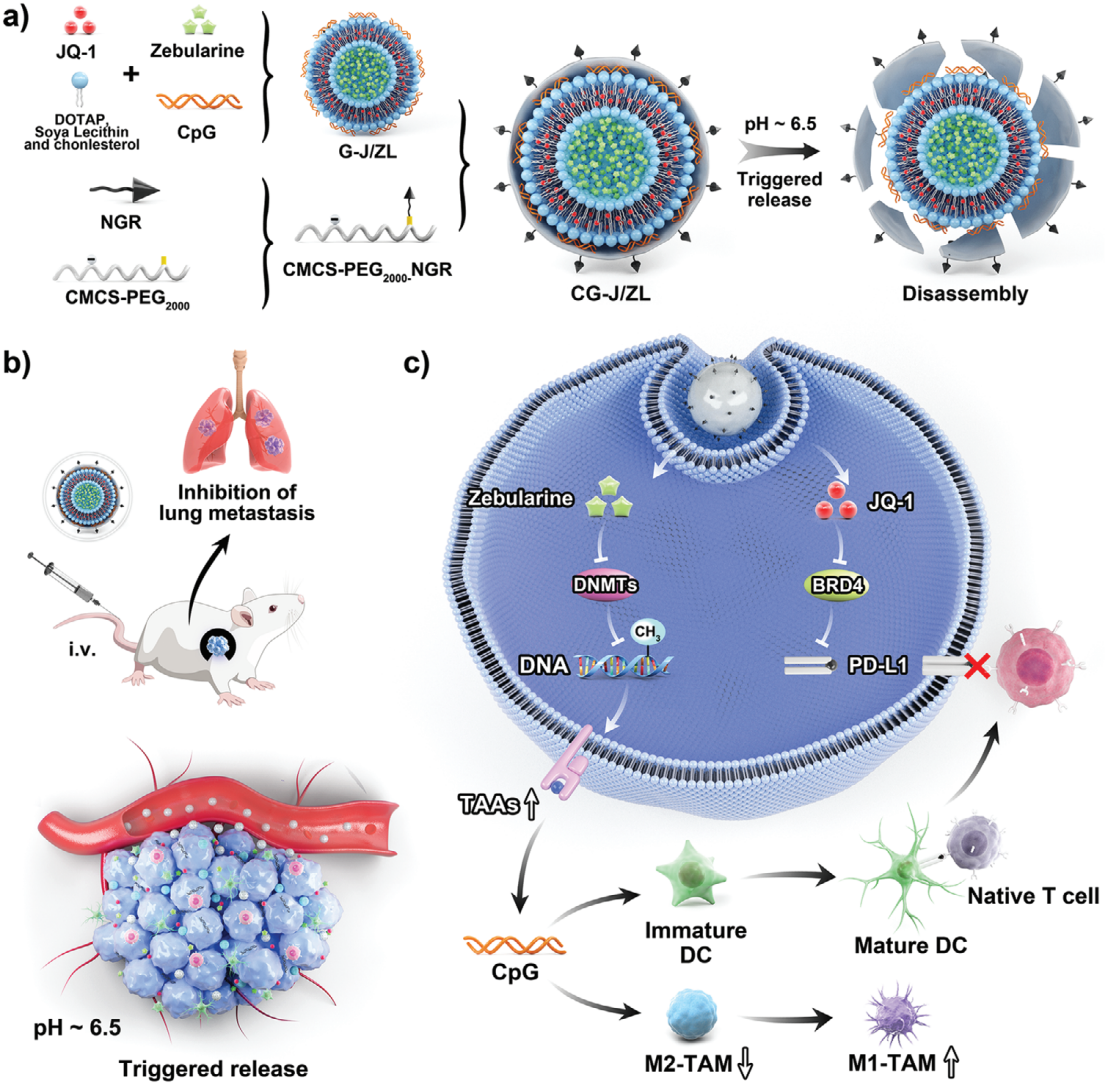
Schematic illustration of the integrated nano‐regulator inhibits tumor immune escape via the “two‐way regulation” epigenetic therapy strategy. a) The assembly and disassembly of the nano‐regulator CG‐J/ZL. b) The “two‐way regulation” function of CG‐J/ZL in vivo. Under the mediation of NGR, CG‐J/ZL could target tumor tissue and trigger disassembly under the acidic TME. Zeb can effectively upregulate TAAs to enhance tumor immunogenicity; JQ1 can inhibit PD‐L1 expression to block immune checkpoint blockade; CpG can promote DC maturation that would cooperate with Zeb to promote activation of T‐cells. Besides, CpG could reactivate the ability of TAM to kill tumor cells.

Herein, the nano‐regulator CG‐J/ZL was successfully constructed. The physicochemical properties and pH‐responsive disassembly of CG‐J/ZL were characterized. Tumor accumulation ability and co‐delivery ability of CG‐J/ZL were investigated. The “two‐way regulation” ability of CG‐J/ZL, including the upregulated TAAs expression, and downregulated PD‐L1 expression were evaluated. In addition, the intratumoral infiltration of T‐cells, DC, TAM, cytotoxic T lymphocytes (CTL), and cytokine secretion were evaluated. We further combined PD‐1 mAb with nano‐regulator CG‐J/ZL to evaluate the antitumor effect and anti‐metastatic efficacy. Collectively, the results showed that CG‐J/ZL could upregulate TAAs expression to enhance T‐cells infiltration and downregulate PD‐L1 expression to improve the recognization of T‐cells to tumor cells, thus inhibiting tumor immune escape, which provides a promising strategy to improve antitumor immune response **Scheme** **1**.

## 2 Results and Discussion

### 2.1 Characterization of the Physicochemical Properties and pH‐Responsive Disassembly of the Nano‐Regulator CG‐J/ZL

**Figure 1:**
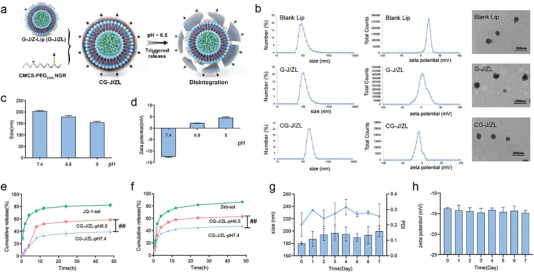
Evaluation of the assembly and disassembly behavior of CG‐J/ZL. a) Scheme of assembly and disassembly behavior for CG‐J/ZL. b) Sizes, zeta potentials and morphologies of Blank Lip, G‐J/ZL, CG‐J/ZL. c) Size and d) zeta potential of CG‐J/ZL at different pH. In vitro release behavior of e) JQ1 and f) Zeb. g) The size, PDI and h) zeta potential of CG‐J/ZL during 7 days (n = 3, ^#^ *p* < 0.05, ^##^ *p* < 0.01, ^###^ *p* < 0.001).

To prepare the nano‐regulator CG‐J/ZL, pH‐responsive and tumor‐targeting material carboxymethyl chitosan–polyethylene glycol–asparagine–glycine–arginine (CPN) was synthesized and evaluated via ^1^H nuclear magnetic resonance (^1^H‐NMR) (Figure S1, Supporting Information). New peaks at 4.25–5.0, at 3.25–3.75, and at 1.4–1.8 ppm were observed in CPN, which attributed to CMCS, PEG and NGR, confirming the successful synthesis of CPN. To engineer the nano‐regulator, Zeb and JQ1 were co‐loaded into the cationic liposomes (J/ZL) by thin‐film dispersion method with condensing CpG via electrostatic interaction to obtain G‐J/ZL. Then, CPN was coated on the surface of G‐J/ZL via electrostatic interaction to construct the core‐shell structure CG‐J/ZL. **Figure** **1**a showed the preparation process and disassembly behavior of CG‐J/ZL. The particle sizes of blank liposome (Blank‐Lip), G‐J/ZL and CG‐J/ZL were 110 ± 1.26, 124.3 ± 3.80, and 190.3 ± 4.3 nm, respectively (Figure 1b). The zeta potentials of Blank Lip, G‐J/ZL and CG‐J/ZL were 20.6 ± 0.46, 5.59 ± 0.56, and −13.5 ± 0.608 mV, respectively. The transmission electron microscopy (TEM) results indicated that Blank Lip, G‐J/ZL and CG‐J/ZL were almost spherical morphology. The encapsulation efficiency (EE%) and drug loading (DL%) of Zeb and JQ1 in G‐J/ZL and CG‐J/ZL were determined. As shown in Table S1 (Supporting Information), the EE% of JQ1 and Zeb in G‐J/ZL was 70.24% ± 2.77% and 16.76% ± 0.54%, respectively. The EE% of JQ1 and Zeb in CG‐J/ZL was 70.88% ± 3.39% and 12.93% ± 3.01%, respectively. The DL% of JQ1 and Zeb in G‐J/ZL was 10.1% ± 0.36% and 1.32% ± 0.046%, respectively. The DL% of JQ1 and Zeb in CG‐J/ZL was 9.74% ± 1.2% and 1.02% ± 0.24%, respectively. Through the above evaluation of physicochemical properties for CG‐J/ZL, we speculated that CG‐J/ZL was successfully prepared.

When CG‐J/ZL reached the acidic TME, the amino group of CPN would be protonated^[^ ^39^ ^]^ leading to the disassembly of CG‐J/ZL and the release of G‐J/ZL. pH‐triggered disassembly characteristics of CG‐J/ZL were observed by DLS. Figure 1c,d showed that the size of CG‐J/ZL decreased from 200 to 150 nm. Besides, the zeta potential of CG‐J/ZL was positively charged at pH 7.4 and negatively charged when pH < 6.8. It suggested that CG‐J/ZL could be disassembled in acidic TME. The pH‐responsive drugs release was evaluated under pH 7.4 and 6.5. As shown in Figure 1e,f, the cumulative release rates of Zeb and JQ1 from CG‐J/ZL at pH 6.5 higher (*p* < 0.001) than those at pH 7.4, further indicating the pH‐responsive property of CG‐J/ZL. In addition, the storage stability of CG‐J/ZL was evaluated via DLS. As shown in Figure 1g,h, the size, polydispersity index (PDI) and zeta potential of CG‐J/ZL did not change significantly during 7 days.

### 2.2 The Tumor Accumulation Ability and Targeting Co‐Delivery Ability of CG‐J/ZL

**Figure 2:**
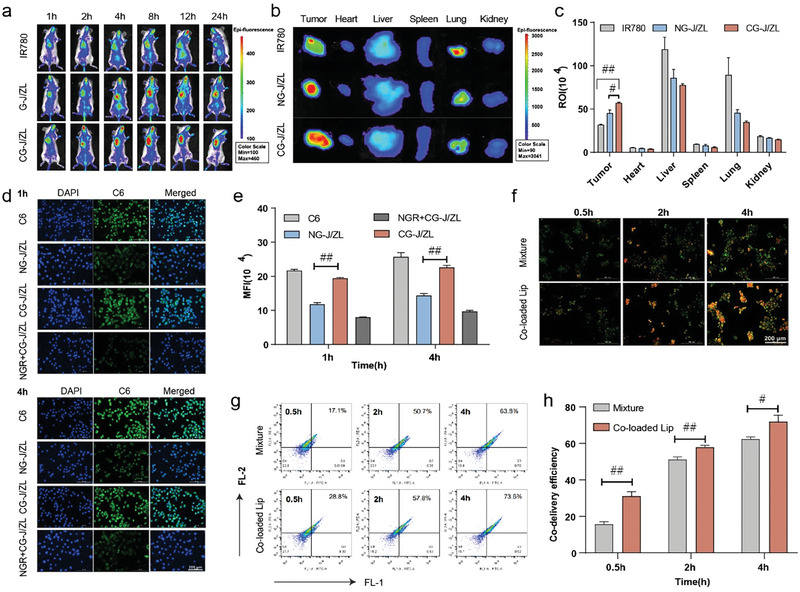
CG‐J/ZL enhanced drugs accumulation at tumor tissue and improved the co‐delivery efficiency of drugs. a) In vivo imaging of 4T1‐bearing mice. b) Ex vivo imaging and (c) total fluorescence intensity of main organs and tumors. d) Fluorescence images and e) flow cytometric analysis of cellular uptake. f–h) flow cytometric analysis of co‐delivery efficiency. (n = 3, ^#^ *p* < 0.05, ^##^ *p* < 0.01, ^###^ *p* < 0.001).

NGR, the ligand of the CD13 receptor expressed on tumor vascular epithelial cells^[^ ^36^, ^40^ ^]^ could effectively promote CG‐J/ZL to accumulate at tumor site. The tumor accumulation capacity of CG‐J/ZL was evaluated via in vivo imaging system (IVIS) in 4T1‐bearing mice. **Figure** **2**a showed that the fluorescence signal intensity of CG‐J/ZL labeled with IR780 at the tumor site was higher than that of the non‐targeted ligand‐modified nano‐regulator NG‐J/ZL labeled with IR780, as well as that of free IR780. The ex vivo images showed that the fluorescence signal intensity of the CG‐J/ZL group at tumor tissues was higher compared with that of the NG‐J/ZL group and free IR780 group (*p* < 0.01) (Figure 2b,c; S2, Supporting Information). The above results indicated that CG‐J/ZL can enhance tumor accumulation ability of drugs under the mediation of NGR. Besides, we evaluated the cellular uptake ability of CG‐J/ZL labeled with coumarin‐6 (C6). Fluorescent images (Figure 2d) and flow cytometric analysis (Figure 2e) showed that the fluorescence signal of CG‐J/ZL was significantly increased compared with that of the NG‐J/ZL group. In addition, the competitive inhibition result indicated that the fluorescence intensity in the CG‐J/ZL group was higher than that of the NGR+CG‐J/ZL group. Subsequently, we evaluated the co‐delivery efficiency of CG‐J/ZL in 4T1 cells. The green C6 and the red Rhodamine B (RhB) were used to replace drugs in the preparation of co‐loaded liposome (co‐loaded lipo), respectively. The yellow fluorescence signal resulting from merging of the red and green fluorescence signals was observed as an indicator of co‐localization efficiency. As shown in Figure 2f–h, the CG‐J/ZL group showed a stronger yellow fluorescence signal than the mixture solution of C6 and RhB, indicating that CG‐J/ZL can effectively co‐deliver different drugs to tumor tissues.

### 2.3 Study on the “Two‐Way Regulation” Function of CG‐J/ZL

**Figure 3:**
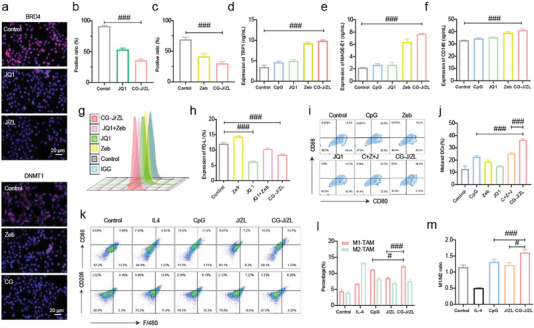
CG‐J/ZL achieved “two‐way regulation” via upregulating TAAs expression and downregulating PD‐L1 expression. a) Immunofluorescence staining analysis of BRD4 and DNMT1 in 4T1 cells. Quantitative analysis of b) BRD4 and c) DNMT1 in 4T1 cells. d–f) ELISA analysis of TAAs expression (TRP1, MAGE‐E1, and CD146) in 4T1 cells. g,h) PD‐L1 expression on 4T1 cells. i,j) BMDC maturation. k,l) The ratio of M1‐TAM and M2‐TAM. m) The ratio of M1‐TAM/M2‐TAM. (n = 3 ^#^ *p* < 0.05, ^##^ *p* < 0.01, ^###^ *p* < 0.001).

Next, the “two‐way regulation” function of CG‐J/ZL, namely the up‐regulation of TAAs and the downregulation of PD‐L1 induced by CG‐J/ZL was investigated. DNMT is involved in DNA methylation, and there are five DNMT that have been identified in mammals including DNMT1, DNMT2, DNMT3A, DNMT3B, and DNMT3L.^[^ ^41^, ^42^ ^]^ Zeb preferentially depletes DNMT1 in tumor cells.^[^ ^43^, ^44^ ^]^ First, we investigated the expression of BRD4 and DNMT1 in 4T1 cells. As shown in **Figure** **3**a, there was a weaker red fluorescence signal in the CG‐J/ZL group compared with the control group. Quantitative analysis of DNMT1 and BRD4 also indicated lower expression of BRD4 and DNMT1 in the CG‐J/ZL group compared with the control group (Figure 3b,c). These results indicated that CG‐J/ZL can decrease the expression of BRD4 and DNMT1. The decreased DNMT1 could inhibit DNA hypermethylation, which would upregulate TAAs expression. Therefore, the expression of TAAs was examined by enzyme‐linked immunosorbent assay (ELISA). The ELISA results of different Zeb concentrations (0, 0.1, 1, 2.5, and 5 µg mL^−1^) incubated with 4T1 cells showed that Zeb could effectively enhance TAAs expression at 2.5 µg mL^−1^ (Figure S3, Supporting Information). Then, 4T1 cells were incubated with various formulations, and the TAAs expression was again analyzed via ELISA. These results showed that Zeb group and CG‐J/ZL group upregulated the expression of MAGE‐E1, TRP1, and CD146 compared with the control group, suggesting that Zeb and CG‐J/ZL could enhance tumor immunogenicity, which could increase the intratumoral infiltration of T‐cells (Figure 3d–f).

Subsequently, we evaluated PD‐L1 expression on 4T1 cells at different JQ1 concentrations (0.1, 0.5, 1, 2.5, 5, 8, and 10 µg mL^−1^) using flow cytometry analysis. The results showed that JQ1 could effectively decrease PD‐L1 expression at 5 µg mL^−1^ (Figure S4, Supporting Information). Then, 4T1 cells were again incubated with the different formulations, and the PD‐L1 expression was analyzed. The results showed that Zeb upregulated PD‐L1 expression compared with the control group, whereas the combination of JQ1 and Zeb antagonized PD‐L1expression. Besides, the CG‐J/ZL group showed downregulation of PD‐L1 expression compared with the control group (Figure 3g,h), which could block PD‐1/PD‐L1 to activate T‐cells to kill tumors. DC, as the most powerful antigen‐presenting cells, can efficiently ingest, process, and present antigens to native T‐cells to regulate T‐cells immunity. CpG can effectively promote DC maturation, which will synergize with Zeb to enhance antigen presentation. Therefore, the in vitro DC maturation was evaluated. Figure 3i,j showed that the matured DC ratio (CD80+ CD86+) in CG‐J/ZL group was 36.57% ± 0.6658%, while only 12.77% ± 2.503% in the control group. As shown in Figure 3k,l, CG‐J/ZL decreased the amount of M2‐TAM and increased the amount of M1‐TAM compared with those of the CpG group. The M1‐TAM/M2‐TAM ratio was also studied and found to be increased in the CG‐J/ZL group compared with the CpG group (Figure 3m), demonstrating that CG‐J/ZL could repolarize M2‐TAM to M1‐TAM.

### 2.4 In Vivo Antitumor Immunity of CG‐J/ZL

**Figure 4:**
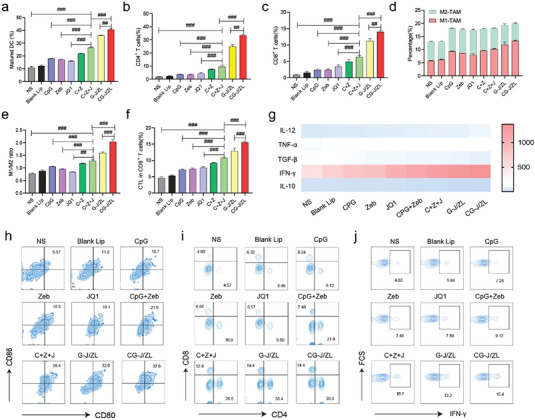
CG‐J/ZL enhanced antitumor immune response in 4T1‐bearing mice. a–c) The percentage of CD4+ T‐cells and CD8+ T‐cells in 4T1 tumor tissues. d) Relative ratio of M1‐TAM and M2‐TAM. e) The M1‐TAM/M2‐TAM ratio. f,g) DC maturation. h,i) Percentage of CTLs infiltrated in 4T1 tumor tissues. j) The levels of cytokines in tumor tissue. (n = 3, ^#^ *p* < 0.05, ^##^ *p* < 0.01, and ^###^ *p* < 0.001).

Previous results have shown that the nano‐regulator CG‐J/ZL can upregulate TAAs expression and downregulate PD‐L1 expression, while promoting DC maturation and repolarizing M2‐TAM to M1‐TAM. Based on the “two‐way regulation” function of nano‐regulator CG‐J/ZL, we speculated that CG‐J/ZL would effectively enhance the intratumoral infiltration of T‐cells and improve the recognization of tumor cells by T‐cells, thereby effectively activating the antitumor immune response. Therefore, the percentage of DC in lymph nodes, the percentage of CD4+T‐cells, CD8+T‐cells, CTL and TAM in tumor tissues and the concentrations of cytokines in tumor tissues were measured. As shown in **Figure** **4**a,h, the mixture of Zeb, CpG, and JQ1 (C+Z+J group) resulted in higher levels of mature DC in lymph nodes compared with the normal saline group (*p* < 0.001), Zeb group (*p* < 0.001), CpG group (*p* < 0.001), and JQ1 group (*p* < 0.001). The CG‐J/ZL group had significantly higher percentages of mature DC than the G‐J/ZL group (*p* < 0.01) or C+Z+J group (*p* < 0.001). The CG‐J/ZL group had higher infiltration of CD4+ T‐cells and CD8+ T‐cells than the G‐J/ZL group (*p* < 0.01) or C+Z+J group (*p* < 0.001) (Figure 4b,c,i). These results indicated that the nano‐regulator CG‐J/ZL can promote antigen presentation and enhance intratumor T‐cells infiltration.

In addition, the percentage of M2‐TAM was decreased in the CG‐J/ZL group compared with other groups (Figure 4d). The M1‐TAM/M2‐TAM ratio in the CG‐J/ZL group was higher than that in the G‐J/ZL group (*p* < 0.001) or C+Z+J group (*p* < 0.001) (Figure 4e), indicating that CG‐J/ZL could repolarize M2‐TAM to M1‐TAM. We further evaluated the percentages of CTL at the tumor site. As shown in Figure 4f,j, the ratio of CTL in the CG‐J/ZL group was higher than those in other groups, indicating that CG‐J/ZL could effectively activate the antitumor immune response. The cytokine levels in the TME were also quantitatively analyzed. As shown in Figure 4g and Figure S5 (Supporting Information), the levels of immunosuppressing cytokines, including IL‐10 and TGF‐β, were lower in the CG‐J/ZL group than in the other groups, whereas levels of immune‐activating cytokines, including IFN‐γ, TNF‐α, and IL‐12, were higher. These results indicated that the nano‐regulator can effectively boost antitumor immunity via the “two‐way regulation” strategy.

### 2.5 In Vivo Anti‐Tumor Efficacy

**Figure 5:**
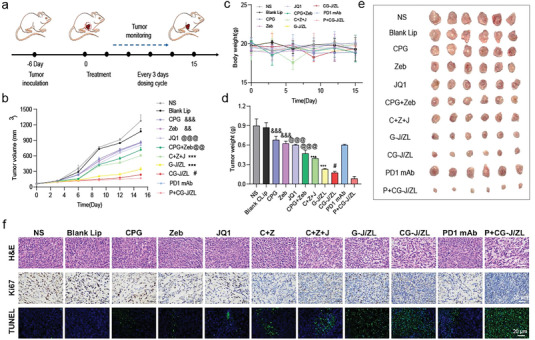
The nano‐regulator CG‐J/ZL enhanced the antitumor efficacy in 4T1‐bearing mice. a) Schedule of in vivo anti‐tumor efficacy. b) Tumor volume and c) body weight of 4T1‐bearing mice. d) Tumor weight and e) tumor images of ex vivo tumors. f) Immunohistochemical images of tumor tissue sections. (n = 6, ^**^ *p* < 0.01, ^***^ *p* < 0.001, compared with CG‐J/ZL. ^#^ *p* < 0.05, compared with P+CG‐J/ZL. ^@^ *p* < 0.05, ^@@^ *p* < 0.01, and ^@@@^ *p*<0.001, compared with C+Z+J. ^&&^ *p* < 0.01, ^&&&^ *p* < 0.001, compared with CpG+Zeb).

The 4T1‐bearing mice were constructed to study the anti‐tumor efficacy of the nano‐regulator CG‐J/ZL. The experiment schedule was shown in **Figure** **5**a. The tumor volumes of 4T1‐bearing mice were measured every two days (Figure S6, Supporting Information). As shown in Figure 5b, Z+J group showed a better tumor inhibition rate than the Zeb group (*p* < 0.01). The tumor inhibition rate of C+Z+J group was higher than CpG+Zeb group (*p* < 0.01), indicating that blocking immune checkpoint could further enhance anti‐tumor efficacy. The tumor inhibition rate of the CG‐J/ZL group was significantly better than C+Z+J group. Of note, compared with the CG‐J/ZL group, the combination of CG‐J/ZL and PD‐1 mAb (P+CG‐J/ZL) further decreased the tumor volume (*p* < 0.001). As shown in Figure 5c, there was no significant change in body weight in the CG‐J/ZL group, indicating low systemic toxicity. Then, tumor tissues were weighed and imaged, which further indicated the excellent antitumor effect of P+CG‐J/ZL (Figure 5d,e). Moreover, hematoxylin and eosin (H&E), Ki67, and TUNEL staining of tumor tissue showed more tumor cell necrosis, less tumor proliferation, and a higher level of apoptosis in the P+CG‐J/ZL group (Figure 5f). These results indicated that combining CG‐J/ZL with PD‐1 mAb significantly improves its antitumor effect.

### 2.6 In Vivo Anti‐Metastasis Effect of CG‐J/ZL

**Figure 6:**
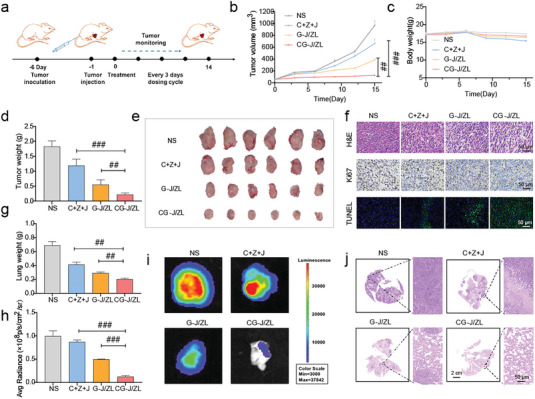
The nano‐regulator CG‐J/ZL exhibited good anti‐metastasis effect. a) The experiment schedule of anti‐metastasis in 4T1‐bearing mice. b) Tumor volume and c) body weight of 4T1 bearing mice. d) Tumor weight and e) tumor photographs. f) Immunohistochemical staining of tumor sections. g) Relative fluorescence intensity of lung. h) Lung weight. i) Ex vivo bioluminescence images of lungs. j) H&E staining images of lungs. (n = 6, ^#^ *p* < 0.05, ^##^ *p* < 0.01, and ^###^ *p* < 0.001).

To further confirm the therapeutic value of CG‐J/ZL, its anti‐metastatic effects in 4T1‐bearing mice were evaluated. The experimental procedure is shown in **Figure** **6**a. As shown in Figure 6b, the orthotopic tumor volume in the CG‐J/ZL group was significantly lower than those of the C+Z+J group (*p* < 0.001) and G‐J/ZL group (*p* < 0.01). Moreover, compared with the normal saline group, there was no significant body weight loss in the CG‐J/ZL group (Figure 6c), indicating the low systemic toxicity of CG‐J/ZL. Tumor tissues were weighted and imaged at the end of treatment, and superior antitumor efficacy was observed in the CG‐J/ZL group (Figure 6d,e). In addition, H&E, Ki67, and TUNEL staining of tumor tissues indicated greater tumor cell necrosis, less tumor proliferation, and better tumor cell apoptosis in the CG‐J/ZL group (Figure 6f). The lungs of mice were also dissected and weighted. Lung weights in the CG‐J/ZL group were lower than those of the C+Z+J group (*p* < 0.01) and G‐J/ZL group (*p* < 0.01) (Figure 6g). The lungs of mice were then dissected, and bioluminescence photographs were obtained via the in vivo imaging system. As shown in Figure 6h,i, the fluorescence intensity of the CG‐J/ZL group was weaker than that of the C+Z+J (*p* < 0.001) or G‐J/ZL (*p* < 0.001) group. To further prove the anti‐metastasis effect of CG‐J/ZL, H&E staining of lung sections was performed. As shown in Figure 6j, the CG‐J/ZL group had fewer metastatic lesions in lung tissues compared with other groups. Besides, the representative in vivo bioluminescence images of mice at day 15 (treatment end) and photo of excised lungs were provided. As shown in the Figure S7 (Supporting Information), CG‐J/ZL group showed weakest fluorescence intensity on the 15th days, indicating suppressed lung metastases in the CG‐J/ZL group. As shown in the Figure S8 (Supporting Information), compared to other groups, CG‐J/ZL showed less lung bloated. The above data suggested that CG‐J/ZL could inhibit the lung metastasis.

We further analysis the immunoreaction in the tumor metastasis model. As shown in Figure S9a–c (Supporting Information), the CG‐J/ZL group induced the highest infiltration of CD4+ T‐cells and CD8+ T‐cells than G‐J/ZL group (*p* < 0.05) and C+Z+J group (*p* < 0.01). Figure S9d,e (Supporting Information) showed that CG‐J/ZL group induced a significantly higher matured DC than G‐J/ZL group (*p* < 0.05) and C+Z+J group (*p* < 0.01). These results indicated that the nano‐regulator CG‐J/ZL can promote antigen presentation and enhance intratumor T‐cells infiltration. We further evaluated the percentage of CTL in tumor site. As shown in Figure S9f,g (Supporting Information), the ratio of CTL in CG‐J/ZL group was higher than other groups, indicating that CG‐J/ZL can effectively activate antitumor immune response.

### 2.7 Preliminary Safety Evaluation of CG‐J/ZL

The preliminary safety of CG‐J/ZL was studied via hemolysis evaluation and H&E staining of the main organs. The images of RBC incubated with CG‐J/ZL showed that no obvious hemolysis of RBC was observed and the hemolysis rates of CG‐J/ZL were <5% (Figure S10, Supporting Information). Besides, H&E staining of the main organs showed that no obvious tissue damage for CG‐J/ZL group was observed (Figure S11, Supporting Information). The above results indicated that CG‐J/ZL exhibited good safety.

## 3 Conclusion

In conclusion, we proposed a “two‐way regulation” epigenetic therapy strategy via engineering a nano‐regulator to inhibit tumor immune escape caused by the low levels of tumor immunogenicity and immune checkpoint‐dependent suppression. The nano‐regulator could upregulate TAAs expression and downregulate PD‐L1 expression. To engineer the nano‐regulator, the DNMT inhibitor Zeb and the BRD4 inhibitor JQ1 were co‐loaded into the cationic liposomes with condensing TLR9 agonist CpG via electrostatic interaction to obtain G‐J/ZL. Then, NGR‐modified CMCS was coated on the surface of G‐J/ZL to construct the core‐shell structure CG‐J/ZL. Under the mediation of NGR, CG‐J/ZL could target tumor tissue and disassemble under the acidic TME. Zeb could effectively upregulate TAAs to improve the immunogenicity by inhibiting DNA hypermethylation; JQ1 could inhibit PD‐L1 expression to block immune checkpoint blockade; CpG could promote DC maturation that would cooperate with Zeb to promote antigen presentation. Besides, CpG could reactivate the ability of TAM to kill tumor cells. Taken together, these results show that the nano‐regulator CG‐J/ZL can upregulate TAA expression to enhance T‐cell infiltration and downregulate PD‐L1 expression to improve the recognition of tumor cells by T‐cells, thereby inhibiting tumor immune escape, a promising strategy to improve antitumor immune response.

## 4 Experimental Section

### Materials

Zeb was obtained from TCI Shanghai Co., Ltd. (Shanghai, China). JQ1 was obtained from Shanghai Macklin Biochemical Co., Ltd. (Shanghai, China). NHS‐PEG_2000_‐NHS was obtained from Ruixi Biological Technology Co., Ltd. (Xi'an, China). CpG was purchased from Shenggong Biotech Co. LTD. (Shanghai, China). CMCS (MW = 50000 Da) was provided by Haidebei Biological Engineering Co., Ltd (Jinan, China). NGR peptide provided by Leon Biological Technology Co. Ltd (Nanjing, China). Enzyme‐linked immunosorbent assay (ELISA) kits of tumor‐associated antigens (MAGE‐E1, CD146, and TRP‐1) were obtained from BoYan Biotechnology Co., Ltd (Nanjing, China). ELISA kits of cytokines (IL‐12, IFN‐γ, TNF‐α, IL‐10, and TGF‐β) were obtained from Dakewe Co., Ltd. (Nanjing, China).

### Cell Lines

Human umbilical vein endothelial cells (HUVEC), mouse breast cancer cell line (4T1), and luciferase‐labeled mouse breast cancer cells (4T1‐Luc) were purchased from the Chinese Academy of Sciences. The above cells were cultured in RPMI‐1640 media with 10% fetal bovine serum.

### Animals

Female BALB/c mice were provided by Beijing Vital River Laboratory Animal Technology Co., Ltd. (Beijing, China). All animal procedures were carried out according to the Animal Management Rules of the Ministry of Health of the People's Republic of China and the Animal Experiment Ethics Review of Shandong University (No.19030).

### Synthesized of CPN

CPN was synthesized according to the previous study^[^ ^45^ ^]^ NGR (12.4 mg), SCM‐PEG_2000_‐SCM (51.2 mg), and DMAP (6.4 mg) were dissolved in 3 mL of PBS (pH 7.4) and stirred for 1.5 h. Then, EDC (8.8 mg) was added to the above solution. 2.5 h later, CMCS (60.0 mg) dissolved in 5 mL of PBS was added to the above solution. 24 h later, the unreacted materials were removed by dialysis (MW = 8–14 kDa) against distilled water. The CPN was obtain after lyophilization and confirmed via ^1^H‐NMR.

### Preparation of J/ZL, G‐J/ZL, CG‐J/ZL, and NG‐J/ZL

DOTAP and soya lecithin (the molar ratio of DOTAP to soya lecithin was 1:20 were dissolved in 2 mL ethanol, evaporated at 40 °C to form the dried lipid film, and hydrated with Zeb solution (4 mg mL^−1^) at 60 °C. Then, the suspension was squeezed for 3 times via membranes filters to get J/ZL. Then CpG was added to J/ZL solution (N/P = 6:1) to obtain G‐J/ZL. The condensation abilities of cationic liposomes were evaluated with agarose gel retardation assay. Finally, CPN (3.6 mg mL^−1^) was added into G‐J/ZL at equal volume and incubated for 0.5 h to obtain the nano‐regulator CG‐J/ZL. The non‐targeted ligand‐modified nano‐regulator was obtained by mixing CMCS and G‐J/ZL at equal volume to get NG‐J/ZL.

### Characterizations of Blank Lip, J/ZL, and CG‐J/ZL

The particle size and size distribution of Blank lipo, J/ZL and CG‐J/ZL were evaluated by DLS. The morphologies of Blank lipo, J/ZL and CG‐J/ZL were assessed by TEM. The DL% and EE% of Zeb and JQ1 were measured by HPLC and calculated by the following:

(1) $$DL\%=\frac{W_{loaded\;drug}}{W_{nano-regulator}}\times 100\%$$

(2) $$EE\%=\frac{W_{loaded\;drug}}{W_{total\;drug}}\times 100\%$$

where *W*_loaded drug_ represented the amount of loaded Zeb and JQ1 in nano‐regulator, *W*_nano‐regulator_ represented the total amount of nano‐regulator, and *W*_total drug_ was the drug added to the nano‐regulator.

### pH‐Responsive Disassembly of CG‐J/ZL

To confirm pH‐responsive disassembly of CG‐J/ZL, CG‐J/ZL was incubated with PBS at different pH (7.4, 6.8, and 5.0) for 0.5 h. Then, the particle size and zeta potential of CG‐J/ZL were evaluated via DLS.

### In Vitro Release of JQ1 and Zeb

In vitro release of JQ1 and Zeb from CG‐J/ZL was evaluated. In brief, 1 mL JQ1 solution, Zeb solution, and CG‐J/ZL were added into dialysis bags (8–14 kDa), which were placed into 15 mL centrifuge tubes. Then, 10 mL PBS with different pH (pH 7.4 and 6.5) was added to centrifuge tubes at 37 °C under horizontal shaking. The released media was collected and replaced with 10 mL fresh media at predetermined time points. The concentration of Zeb and JQ1 was studied via HPLC.

### Hemolysis Assay

The red blood cells (RBC) suspension that obtained from rat were washed and collected by centrifugation. CG‐J/ZL with different Zeb concentrations (Zeb: 20, 40, 80, 100, and 120 µg mL^−1^) was added into RBC suspension and incubated at 37  C for 3 h. After centrifuging, the absorbance of supernatant was determined via UV–vis spectrophotometer (576 nm).

### Evaluation of Active Targeting and Tumor Accumulation

The active targeting ability of CG‐J/ZL was evaluated on HUVEC. C6 was used to replace Zeb and JQ1. HUVEC were inoculated in 12‐well plate (1.0 × 10^5^ cells per well). 12 h later, CG‐J/ZL labeled with C6, NG‐J/ZL labeled with C6, and free C6 were added and incubated for 1 and 4 h. Besides, HUVEC were preincubated with free NGR (1 mg mL^−1^) for 1 h, then CG‐J/ZL labeled with C6 was added for competitive inhibition experiments. HUVEC were stained with DAPI and evaluated by fluorescence images and flow cytometer. The tumor accumulation of CG‐J/ZL was evaluated on 4T1 bearing BALB/c via IVIS, and Zeb and JQ1 were replaced with IR780. When the tumor grew for 14 days, mice were randomized into three groups: 1) IR780, 2) NG‐J/ZL labeled with IR780, and 2) CG‐J/ZL labeled with IR780. Mice, tumor tissues and major organs were observed via IVIS.

### Co‐Delivery Efficiency of CG‐J/ZL

Drugs in CG‐J/ZL were replaced with C6 and RhB, respectively. 4T1 cells were seeded in 12‐well plates. 12 h later, the mixture solution of C6 and RhB, and co‐loaded liposome were added. 2 h later, the cells were evaluated via fluorescence images and flow cytometer (Accuri C6 Plus, BD, USA).

### Evaluation of TAAs Expression on 4T1

To evaluate TAAs expression (MAGE‐E1, CD146, and TRP‐1), 4T1 cells were inoculated in 12‐well plates and cultured overnight. Zeb, JQ1, CpG and CG‐J/ZL were added. 24 h later, the level of cytokines according to the operation instructions of ELISA kits.

### Evaluation PD‐L1 Expression of on 4T1

4T1 cells were seeded in 12‐well plates and grown overnight. Zeb, JQ1, Zeb+JQ1, and CG‐J/ZL were added for 24 h. Then, the cells were incubated with PD‐L1 mAb and anti‐mouse IgG/Alexa Fluor 488 goat antibody and measured by flow cytometry.

### In Vitro DC Maturation

BMDC was incubated with granulocyte–macrophage colony‐stimulating factor (GM‐CSF) and interleukin‐4 (IL‐4) for 6 days. Then CpG, Zeb, JQ1, C+Z+J, and CG‐J/ZL was added. 24 h later, the BMDC was labeled with different antibodies and determined by flow cytometry.

### Evaluation of In Vitro TAM Polarization

RAW264.7 cells were seeded into 12‐well plates for 12 h. IL‐4 (15 ng mL^−1^) was added. 12 h later, fresh 1640 medium containing CpG solution, J/ZL, and CG‐J/ZL (CpG: 1.5 µg mL^−1^) were added for 24 h. Then, RAW264.7 was labeled with antibodies and measured by flow cytometer.

### Evaluation of In Vivo Antitumor Effect

4T1‐bearing mice were established to evaluate anti‐tumor effect. Briefly, 4T1 cells were injected to the right mammary grand of the female BALB/c mice. When the tumor volume grew to nearly 100 mm^3^ mice were randomized into 11 groups, which treated with various formulations every 3 days as following: 1) normal saline (NS), 2) Blank Lip, 3) CpG, 4) Zeb, 5) JQ1, 6) CpG+Zeb, 7) C+Z+J, 8) G‐J/ZL, 9) CG‐J/ZL, 10) PD‐1 mAb, 11) P+CG‐J/ZL. The dose of Zeb, JQ1, and CpG was 5.0, 10.0, and 2.5 mg k^−1^g, respectively. The dose of PD‐1 mAb was 5 mg k^−1^g and intraperitoneally injected into BALB/c mice. The tumor volumes and body weights were measured every 3rd day. On the 15th day, the mice were sacrificed, and tumor tissue, heart, liver, spleen, lung, and kidney of mice were collected. Tumor tissues were stained with H&E, Ki67, and TUNEL. Main organs were stained with H&E.

### Evaluation of CG‐J/ZL Facilitated Antitumor Immunity

The lymphocytes including CD4+ T‐cells, CD8+ T‐cells, and CTL, TAM were obtained via mincing tumor tissues, filtering through copper network, and centrifuging, then labeled with different antibodies. To evaluate matured DC, lymph nodes were harvested to obtain single‐cell suspensions, then labeled with different antibodies. Besides, tumor cell homogenates were obtained to measure cytokines via ELISA kits (Dakewe, Nanjing, China).

### In Vivo Anti‐Metastasis Evaluation

4T1 cells were injected into the left mammary fat pad of BALB/c female mice. Luc‐4T1 cells were injected into 4T1 bearing mice intravenously to construct the metastatic tumor model. Mice were randomly divided into four groups to treat every three days for 5 times, including (1) NS, (2) C+Z+J, (3) G‐J/ZL, (4) CG‐J/ZL. The dose of Zeb, JQ1, and CpG was 5.0, 10.0, and 2.5 mg k^−1^g, respectively. 15 days later, the lung metastasis of the mice treated with different formulations was evaluated via IVIS. Next, the tumor tissues and lungs were isolated to weigh and photograph. The lungs were harvested with 4% formaldehyde solution, and then stained with H&E.

### Statistical Analysis

Statistical significances were performed by Student's *t*‐tests and One‐way analysis of variance (ANOVA).

## Conflict of Interest

The authors declare no conflict of interest.

## Supporting information

Supporting Information

## Data Availability

The data that support the findings of this study are available on request from the corresponding author. The data are not publicly available due to privacy or ethical restrictions.
