# Supplementary material for: Nano‐Regulator Inhibits Tumor Immune Escape via the “Two‐Way Regulation” Epigenetic Therapy Strategy
Source: Adv Sci (Weinh). 2023 Dec 18;11(9):2305275. doi: 10.1002/advs.202305275 (PMC10916662; doi:10.1002/advs.202305275)
Supplement: Supplementary file 1 — Supporting Information [file ADVS-11-2305275-s001.pdf]

ADVANCED SCIENCE
Open Access

Supporting Information

for *Adv. Sci.*, DOI 10.1002/advs.202305275

Nano-Regulator Inhibits Tumor Immune Escape via the "Two-Way Regulation" Epigenetic Therapy Strategy

Shuang Liang, Meichen Liu, Weiwei Mu, Tong Gao, Shuying Gao, Shunli Fu, Shijun Yuan, Jinhu Liu, Yongjun Liu, Dandan Jiang and Na Zhang**

Supporting Information

Nano-regulator inhibits tumor immune escape via the "two-way regulation" epigenetic therapy strategy

Shuang Liang[1‡], *Meichen Liu*[1‡] *Weiwei Mu*[1], *Tong Gao*[1], *Shunli Fu*[1], *Shijun Yuan*[1], *Jinhu Liu*[1], *Yongjun Liu*[1], *Dandan Jiang*[2]*, *and Na Zhang*[1]*

Table S1. The encapsulation efficiency and drug loading of JQ1 and Zeb in C-JZL and CG-JZL.

	**EE%**		**DL%**	
**Formulations**	JQ1	Zeb	JQ1	Zeb
**G-JZL**	70.24±2.77	16.76±0.54	10.1±0.36	1.32±0.046
**CG-JZL**	70.88±3.39	12.93±3.01	9.74±1.20	1.02±0.24

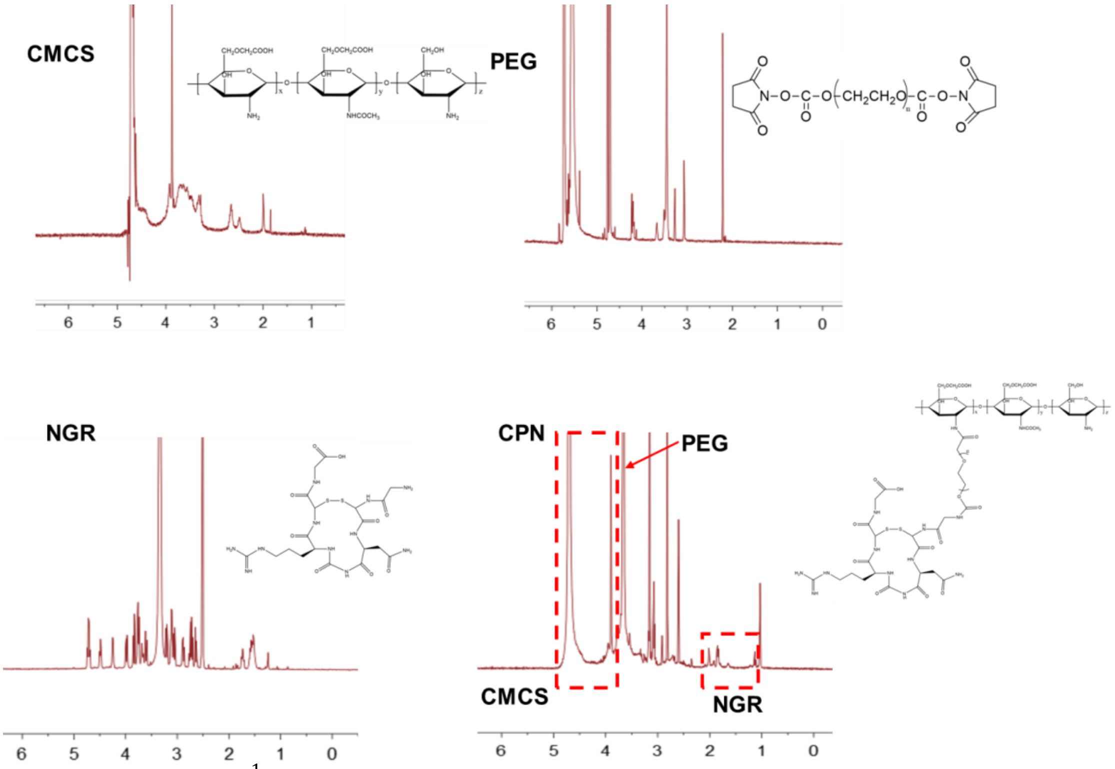

Figure S1. [1] H NMR spectra of NGR, NHS-PEG-NHS, CMCS and CPN.

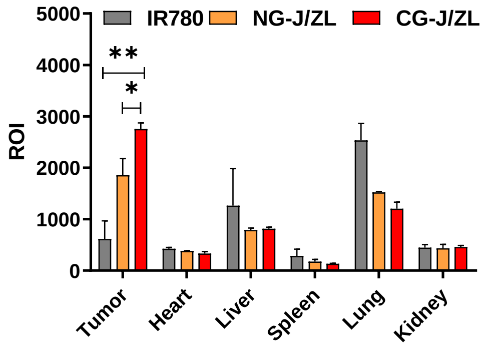

Figure S2. Average fluorescence intensity of main organs and tumors.

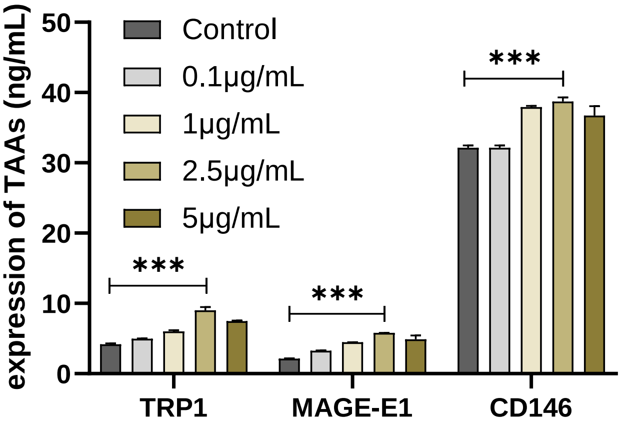

Figure S3. ELISA analysis of TAAs expression at different Zeb concentrations on 4T1 cells.

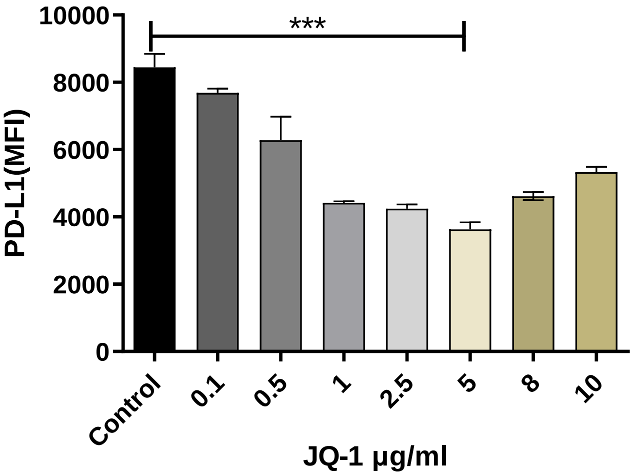

Figure S4. Flow cytometric analysis of the expression of PD-L1 at different JQ1 concentrations on 4T1 cells.

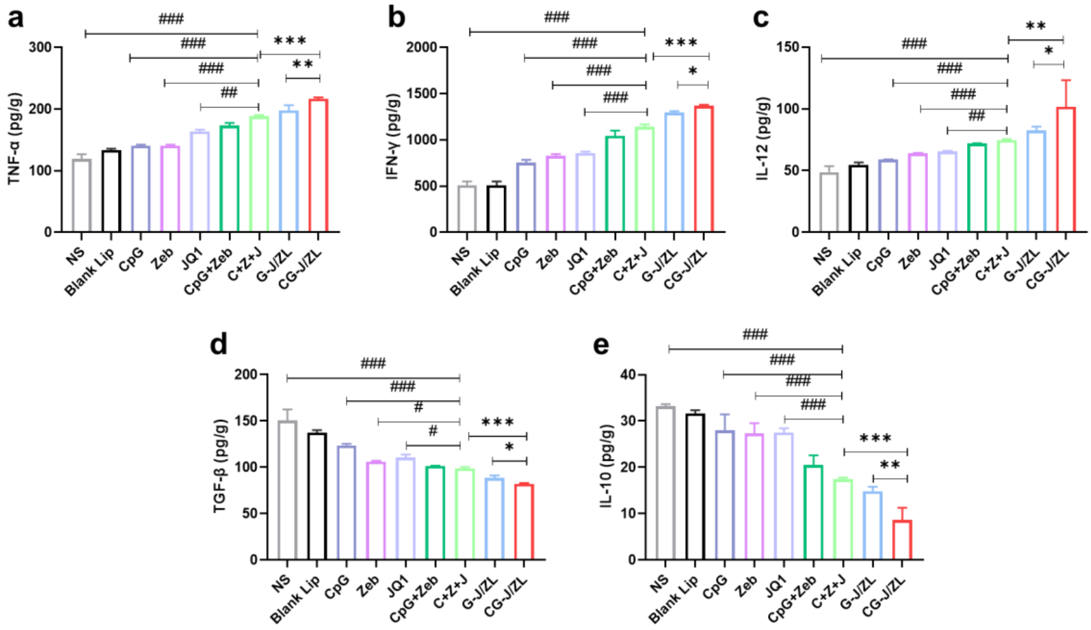

Figure S5. The levels of cytokines s including (a) TNF-α, (b) IFN-γ, (c) IL-12, (d) TGF-β and (e) IL-10 secretion in tumor tissue.*p < 0.05, **p< 0.01, ***p < 0.001, compared with CG-J/ZL group; #p < 0.05, ##p < 0.01, ###p < 0.001 compared with C+Z+J group.

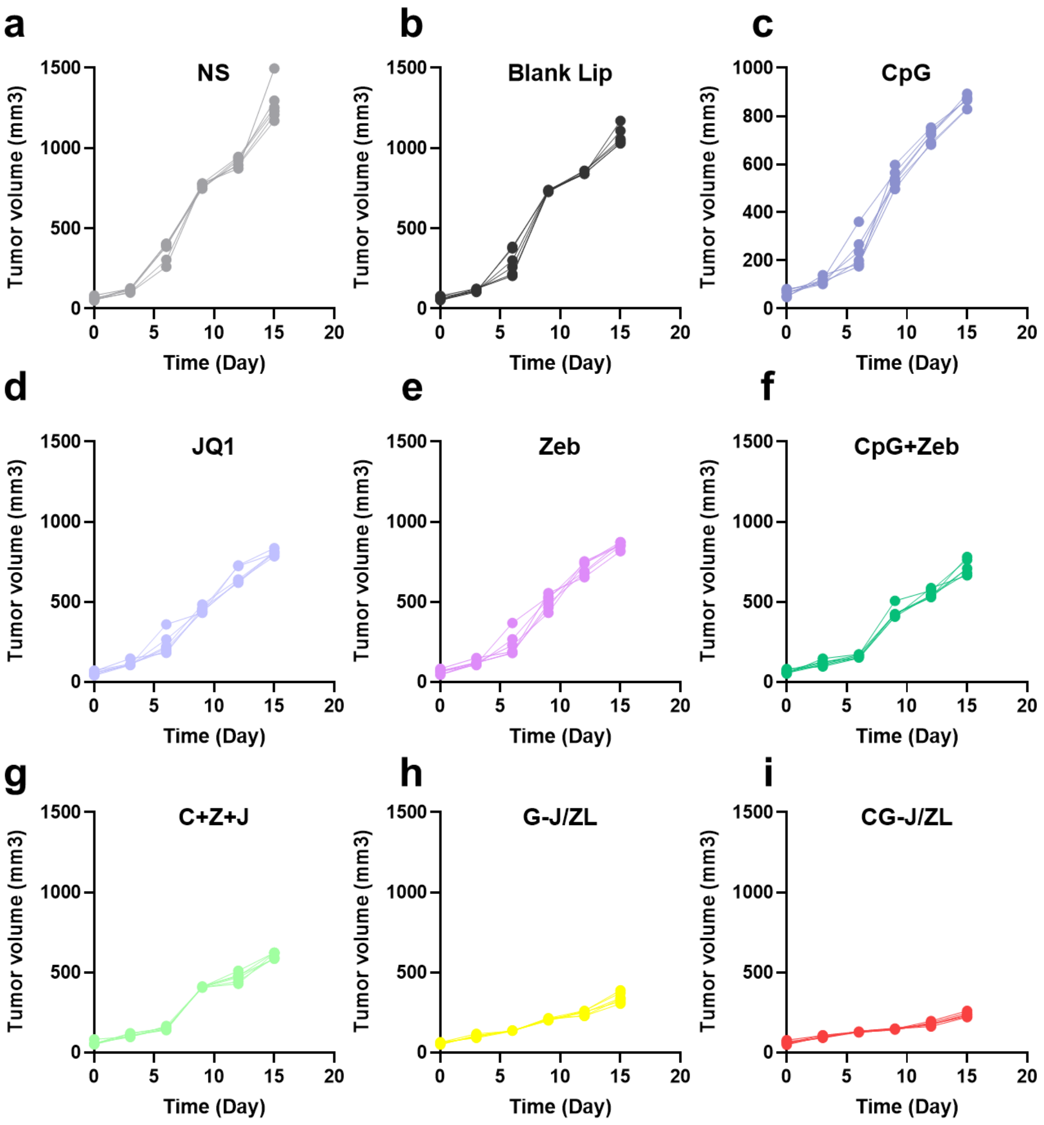

Figure S6. Individual tumor growth curves of 4T1 bearing mice after treated with (a) NS, (b) Blank Lip, (c) CpG, (d) JQ1, (e) Zeb, (f) CpG + Zeb, (g) C+Z+J, (h) G-J/ZL, (i) CG-J/ZL.

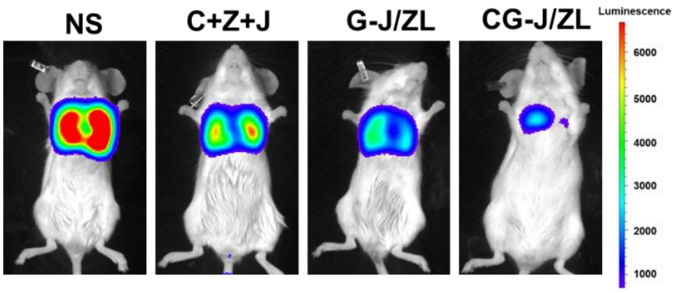

Figure S7. Representative *in vivo* bioluminescence images of mice at day 15 (treatment end) for four therapeutic groups

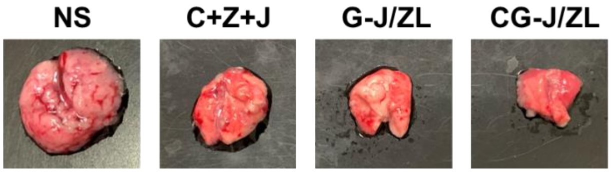

Figure S8. Excised lungs from mice for four therapeutic groups

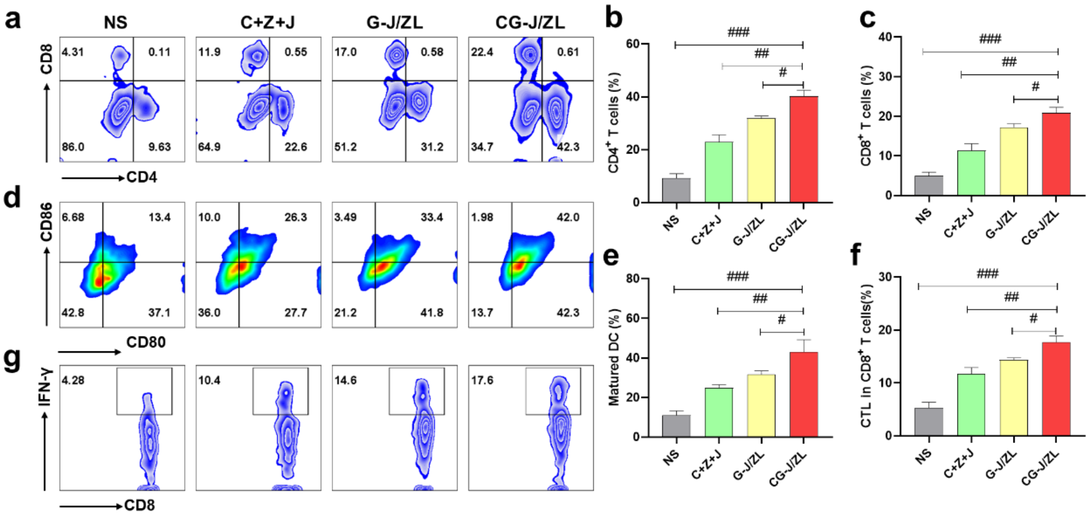

Figure S9. CG-J/ZL enhanced antitumor immune response in lung metastasis model of 4T1-bearing mice. a-c) The percentage of CD4+ T cells and CD8+ T cells in 4T1 tumor tissues. d-e) DC maturation. f-g) Percentage of CTLs infiltrated in 4T1 tumor tissues. (n=3, $^{\#}p$<0.05, $^{\#\#}p$<0.01 and $^{\#\#\#}p$<0.001).

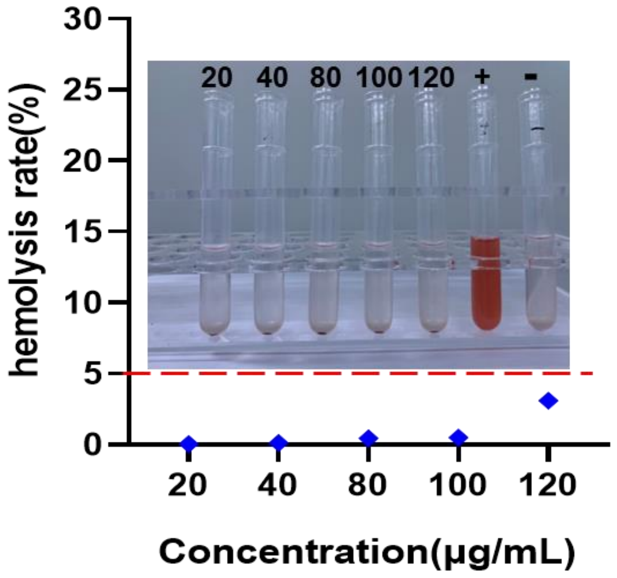

Figure S10. Image and hemolysis rate of RBC after RBC suspension was incubated with CG-J/ZL at different concentration.

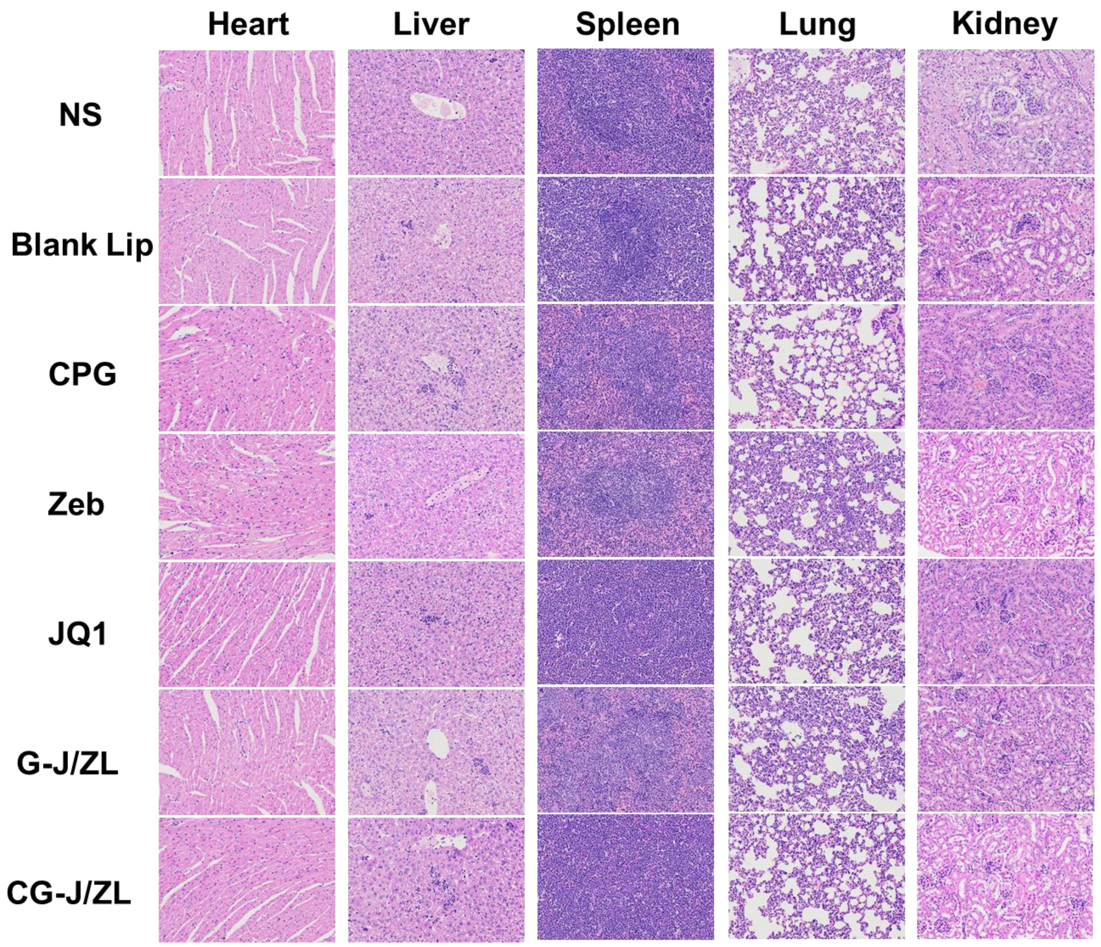

Figure S11. H&E staining images of histological sections. (scale bar=400)
